# Evaluation of aesthetic and functional outcomes in rhinoplasty surgery: a prospective study^☆^

**DOI:** 10.1016/j.bjorl.2016.06.010

**Published:** 2016-07-20

**Authors:** Sara Sena Esteves, Miguel Gonçalves Ferreira, João Carvalho Almeida, José Abrunhosa, Cecília Almeida e Sousa

**Affiliations:** Centro Hospitalar do Porto, Departamento de Otorrinolaringologia, Porto, Portugal

**Keywords:** Rhinoplasty, Aesthetics, Patient satisfaction, Questionnaire, Outcomes evaluation, Rinoplastia, Estética, Satisfação do paciente, Questionário, Avaliação dos desfechos

## Abstract

**Introduction:**

Evaluation of surgery outcome measured by patient satisfaction or quality of life is very important, especially in plastic surgery. There is increasing interest in self-reporting outcomes evaluation in plastic surgery.

**Objective:**

The aim of our study was to determine patient satisfaction in regard to nose appearance and function with the use of a validated questionnaire, before and after rhinoplasty surgery.

**Methods:**

A prospective study was realized at a tertiary centre. All rhinoplasty surgeries performed in adults between February 2013 and August 2014 were included. Many patients underwent additional nasal surgery such as septoplasty or turbinoplasty. The surgical procedures and patients’ characteristics were also recorded.

**Results:**

Among 113 patients, 107 completed the questionnaires and the follow-up period. Analysis of pre-operative and post-operative Rhinoplasty Evaluation Outcome showed a significant improvement after 3 and 6 months in functional and aesthetic questions (*p* < 0.01). In the pre-operative, patients anxious and insecure had a worse score (*p* < 0.05). Difference in improvement of scores was not significant when groups were divided on basis of other nasal procedures, primary or revision surgery and open versus closed approach.

**Conclusion:**

We found that patients with lower literacy degree were more satisfied with the procedure. Rhinoplasty surgery significantly improved patient quality of life regarding nose function and appearance.

## Resumo

**Introdução:**

A avaliação do desfecho de cirurgia medido pela satisfação ou qualidade de vida do paciente é muito importante, especialmente em cirurgia plástica. Existe um interesse crescente na autoavaliação de desfechos nesta especialidade cirúrgica.

**Objetivo:**

O objetivo deste estudo foi determinar a satisfação do paciente em relação à aparência e função do nariz com o uso de um questionário validado, antes e depois da cirurgia de rinoplastia.

**Método:**

Estudo prospectivo realizado em um centro terciário. Todas as cirurgias de rinoplastia realizadas em adultos entre fevereiro de 2013 e agosto de 2014 foram incluídas. Muitos pacientes foram submetidos à cirurgia nasal adicional, como septoplastia ou turbinoplastia. Os procedimentos cirúrgicos e as características dos pacientes também foram registrados.

**Resultados:**

Entre 113 pacientes, 107 completaram os questionários e o período de acompanhamento. A análise da avaliação do desfecho de rinoplastia (ADR) no pré-operatório e pós-operatório mostrou uma melhora significativa após 3 e 6 meses em questões funcionais e estéticas (*p* < 0,01). No pré-operatório, os pacientes ansiosos e inseguros apresentaram um escore pior (*p* < 0,05). A diferença na melhoria dos escores não foi significativa quando os grupos foram divididos com base em outros procedimentos nasais, cirurgia primária ou revisão e abordagem aberta versus fechada.

**Conclusão:**

Verificou-se que pacientes com menor grau de alfabetização estavam mais satisfeitos com o procedimento. A cirurgia de rinoplastia melhorou significativamente a qualidade de vida do paciente quanto à função e aspecto do nariz.

## Introduction

Evaluation of surgery outcome measured by patient satisfaction or quality of life is very important, especially in plastic surgery. There are many areas in otolaryngology in which outcomes are being evaluated such as head and neck oncology, acute sinusitis and obstructive sleep apnea. There is increasing interest in self-reporting outcomes evaluation in plastic surgery, being the facial plastic surgery one of the most important areas of research. Outcomes of any surgical procedure can be measured by quantitative and/or qualitative terms. In the case of plastic surgery, the procedures are generally elective and undertaken for cosmetic purposes, and as such analysis of quantitative parameters like days of internment, morbidity and mortality may be applicable but are not relevant. Therefore, facial plastic surgeons measure success based on qualitative evaluations. However, the lack of a standardized qualitative assessment makes it difficult to compare objectively the success of different techniques and individual surgeons.

Self-reporting on outcomes is increasingly recognized as an important outcome in clinical trials or to assess the effectiveness of medical procedures. Therefore, questionnaires designed to evaluate quality of life and self-image are very helpful in assessing the success of facial plastic surgery as they standardize the collected information and allow objective comparison of procedures by measuring positive and negative effects as well as improvements after rhinoplasty.^1^, ^2^, ^3^ Patient satisfaction depends on subjective factors such as patient perception of pre-operative appearance, patient expectations, social relationship capacities, alcohol intake and temperament.^4^ Compared with primary rhinoplasty, revision rhinoplasty is a more challenging surgery because its main goal is to correct the functional and/or cosmetic defects or complaints after the previous surgery failed to meet patient expectations.^5^ Therefore, understanding patient expectations pre-operatively is crucial to achieve the desired outcomes.^6^ Surgeon and patient are generally not similarly pleased with the procedure, since the expectations and opinions are different.

**Figure 1:**
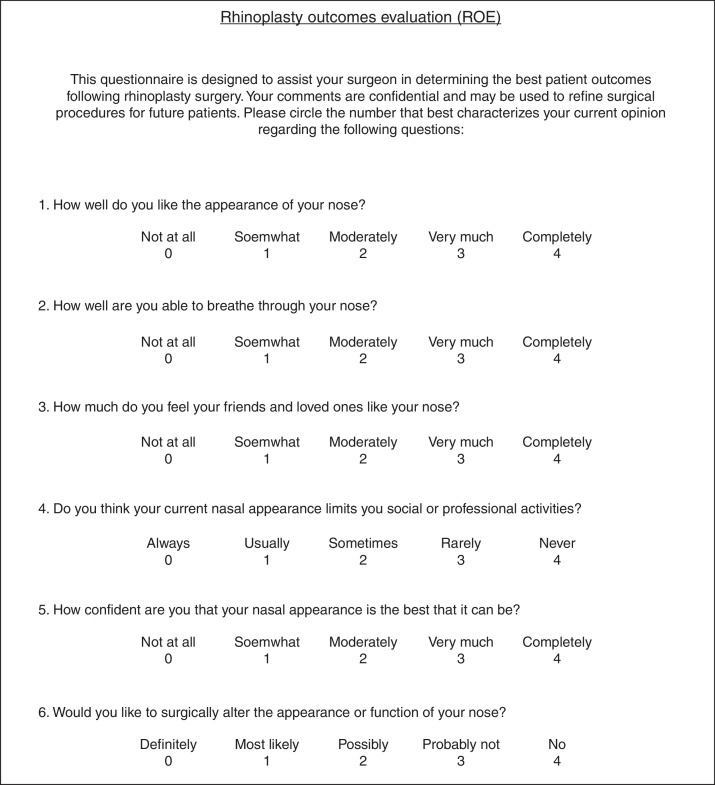
English version of Rhinoplasty Outcomes Evaluation questionnaire.

In 2000, Alsarraf et al. were the first to create and test a questionnaire with reliability, internal consistency and validity for several plastic surgeries, including rhinoplasty.^7^, ^8^ This questionnaire, the Rhinoplasty Outcomes Evaluation (ROE), allowed measure of qualitative aspects such as social, emotional and psychological variables (Fig. 1). In Portugal the study of patient satisfaction after rhinoplasty has been a neglected area mainly due to absence of validated instruments to assess the objective and subjective outcomes of the procedure. In 2013, Sena Esteves et al. validated this ROE questionnaire to Portuguese.^9^

The aim of this study was to evaluate the satisfaction of patients who underwent rhinoplasty in a tertiary centre using the ROE questionnaire pre-operatively and post-operatively and determine the relation with patient characteristics and surgery details.

## Methods

The Research Ethics Committee of our hospital approved the study priors to initiation (study ID: 051/13). We performed a prospective study of all adults that underwent rhinoplasty between February 2013 and August 2014 in a tertiary centre. We identified 110 patients but 3 patients were omitted from the study as they were unresponsive to repeated phone calls.

We included 107 patients who underwent a pre-operative consultation with an otolaryngologist and answered the ROE questionnaire. In addition, the questionnaires asked patient demographic data such as age, sex, ethnicity, literacy level, psychological aspects, reason for visit and research about aesthetic surgery before the consultation. Post-operative satisfaction was evaluated by a phone call at 3 and 6 months after surgery, by the same otolaryngologist. The person making the phone call was not necessarily the surgeon. Patients were also asked if they would still choose to undergo rhinoplasty, knowing the final result.

**Figure 2:**
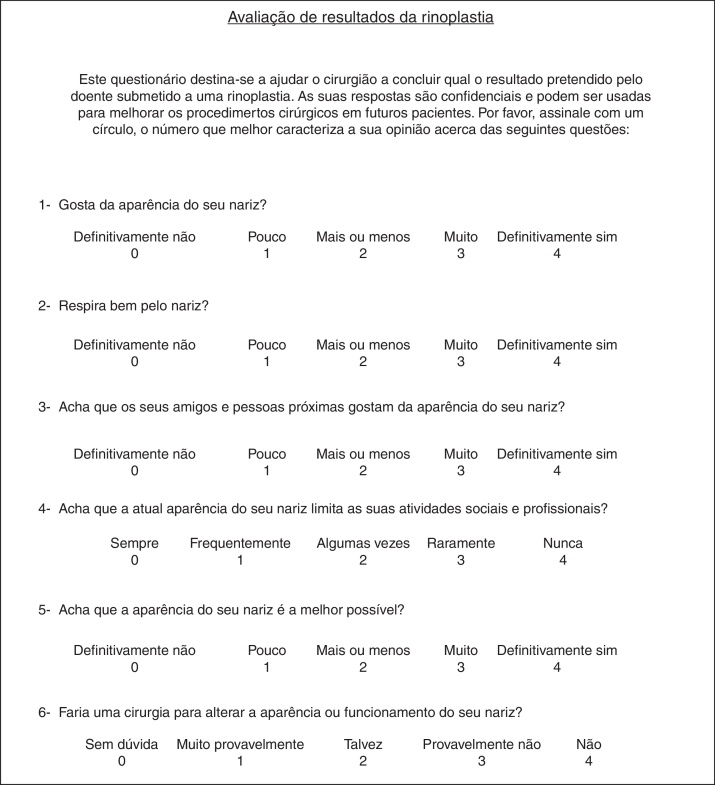
Portuguese version of Rhinoplasty Outcomes Evaluation questionnaire.

The validated Portuguese version of the ROE questionnaire was used and it is composed of six questions (5 about nose shape and 1 about nasal breathing). Each question is scored by the patient on a scale from 0 to 4, where 0 is the most negative answer and 4 the most positive one (Fig. 2). The sum of the scores was divided by 24 and multiplied by 100 to obtain a result ranged from 0 to 100. A lower score indicates more dissatisfaction. A positive difference between post-operative and pre-operative scores means improvement after intervention.

Surgeons also answer a questionnaire, in the day of the surgery, about operative techniques used during the rhinoplasty, other nasal procedures and previous rhinoplasty.

All patients seeking rhinoplasty, even in addition to other nasal procedures such as septoplasty or turbinoplasty were included in the study. Patients younger than 18 years and with congenital or neoplastic nasal deformities were excluded.

The follow-up time was at least 12 months.

Data analysis was done with IBM SPSS Statistic 20 software. Two-tailed *t*-test and one way Anova test in a specific situation (evaluation of literacy degree and satisfaction) were used to analyze data. A *p* < 0.05 was considered statistically significant.

## Results

After inclusion and exclusion criteria were met, 107 patients participated is this study. The sample was composed of 56 female and 51 male patients. The population was divided into three groups: 18–29 years old, 30–49 years old and ≥50 years old. Demographics characteristics of the patients are detailed in Table 1.

**Table 1:** Patient characteristics.

	No. of patients	%
*Gender*		
Male	51	47.7%
Female	56	52.3%

*Age (years)*		
18–29	47	43.9%
30–49	54	50.5%
≥50	6	5.6%

*Ethnicity*		
Caucasian	107	100.0%

*Literacy degree*		
Below 9th grade	9	8.4%
9th grade	25	23.4%
12th grade	42	39.3%
College degree	21	19.6%
MSc/PhD	10	9.3%

*Reason for surgery*		
Functional	14	13.1%
Aesthetic	5	4.7%
Both	88	82.2%

The reasons for undergoing rhinoplasty were aesthetic in 4.7% of patients, functional in 13.1% and a combination of aesthetic and functional in 82.2%. The majority of patients researched information about plastic surgery and rhinoplasty before the first otolaryngology consultation (57%). When we asked in the pre-operative consultation if they had an ideal nose that they would like to transpose to them, 96% answered no and 11% answered yes.

It was also asked if the surgeon explained what would be corrected in the nose surgery and 2% answered not at all, 10% answered somewhat, 25% answered moderately, 31% answered very much and 39% answered completely.

Regarding psychological aspects, 59% of the patients considered themselves anxious and 78% secure (Table 2).

**Table 2:** Psychological characteristics.

	Insecure	Secure	Total
Anxious	23 (21.5%)	36 (33.6%)	59 (55.1%)
Calm	6 (5.6%)	42 (39.3%)	48 (44.9%)
Total	29 (27.1%)	78 (72.9%)	107 (100.0%)

The mean ROE pre-operatively score was 32.8 ± 12.1(range 8.3–58.3) and the mean score post-operatively was 81.2 ± 17.9 at 3 months (range 25–100) and 81.9 ± 17.1 at 6 months (range 37.5–100). Statistical analysis of ROE scores showed significant improvement from pre-operative to post-operative period (*p* < 0.05). However, there was no difference between 3 and 6 months’ post-operative scores.

The correlation between psychological aspects and satisfaction is presented in Table 3, showing that anxious patients were significantly less satisfied than calm patients in the pre-operative period.

**Table 3:** Mean pre- and post-operative scores and correlation with psychological aspects.

	Pre-operatively	Post-operatively (3 months)	Post-operatively (6 months)
*Anxious*			
Mean	30.72	80.22	81.28
SD	11.71	17.81	17.16

*Calm*			
Mean	35.33	82.38	82.46
SD	12.24	18.12	17.12

*p*-Value	0.050	0.539	0.724

SD, standard deviation.

There were no gender differences in mean post-operative scores (*p* > 0.05), but the mean post-operative score of patients with higher literacy degree were lower, indicating less satisfaction (*p* < 0.05).

Primary rhinoplasty was performed in 87.9% and revision rhinoplasty in 12.1% patients. There was no significant difference in post-operative ROE scores between the two groups.

We evaluated the different surgical approaches used and the concomitant nasal procedures. The surgical approaches used were open rhinoplasty (*n* = 27), delivery approach (*n* = 35) and non-delivery approach (*n* = 45). We found no significant difference in ROE score improvement between open and closed technique (*p* = 0.765) or between the two closed techniques (*p* = 0.071).

There were 91 patients who underwent septoplasty, 88 turbinoplasty and 11 functional endoscopic sinus surgery. ROE score after surgery was not significant between these groups (*p* > 0.05).

When asked if they would undergo the surgery again knowing the final result they would undergo surgery: 72.9% answered definitely, 11.2% probably, 6.5% maybe, 5.6% probably not and 3.7% not at all.

Finally, it is important to note that 100% of patients had a higher ROE score after surgery, showing that all patients were more satisfied after the rhinoplasty.

## Discussion

Rhinoplasty is among the most common surgeries performed by facial plastic surgeons worldwide.^10^

This procedure has low patient satisfaction compared with other cosmetic surgeries.^11^ Patient satisfaction is the principal outcome measure of success in facial cosmetic surgeries, yet most surgeons do not use quantitative tools to access it. Patient's satisfaction may be influenced by social environment, education, life experience and level of expectations, which may or may not be realistic. Complete photographic documentation is fundamental to both physician and patients for surgery planning and assessment of post-operative results.^12^ In the present study we chose to use the ROE questionnaire because it was validated by us in Portugal.^9^ This questionnaire quantifies the result from the surgical procedure, assessing respiratory function, quality of life and cosmetic results. Surgeon goal is to improve both cosmetic and breathing, and not purely the aesthetic component.

In this prospective study with 107 patients we evaluate ROE score before surgery and at 3 and 6 months later, allowing more precise results about satisfaction.

Our results showed statistically significant improvement in ROE scores after rhinoplasty, demonstrating a high index of satisfaction in this patient population. Interestingly, the change in ROE scores was higher in lower literacy patients, which may be explained by lower pre-surgical expectations and lack of information and internet access. Sex, age, primary versus revision surgery and additional nasal procedures such as septoplasty, turbinoplasty or FESS showed no significant differences in ROE scores. Of note, the surgical technique (open or closed) has no effect on the ROE scores after rhinoplasty showing the outcome of the surgery was the same regardless the surgical approach used.

In our study the mean pre-operative ROE score was 32.78 and the mean improvement was 49.03 after surgery. These numbers are in line with those reported by Alsarraf et al., which found a mean pre-operative score of 38.8 and a mean improvement of 44.5.^8^ Although the significant improvement in ROE scores in our population, only 72.9% would definitely choose to undergo the same procedure again.

This study focused on a patient population from a public hospital, where rhinoplasty is performed in association with other nasal procedures and doctors who do pre-operative consultations are more or less experienced in those fields. This can be the reason for 12% of patients that answered not at all or somewhat, not having a clear understanding of the surgeon explanation about the proposed aesthetic surgery. Ideally, all patients should be clarified about the proposed surgery.

In our sample there were 51 men and 56 women, which shows that men are increasingly concerned about their physical appearance.

Prospective studies are really important since they permit to choose good candidates for surgery and to assess objectively surgery results.

This study was conducted in an otolaryngology department of a central public hospital composed of senior specialists and residents. One limitation of the study is the fact of rhinoplasty being performed by different surgeons with different levels of experience in the aesthetic area. Therefore, both expectations created in the pre-operative consultation and the post-operative satisfaction survey may be affected by these conditions.

## Conclusions

We conclude the ROE questionnaire is a useful tool for evaluating outcomes of rhinoplasty surgery. Our patient's satisfaction at 3 and 6 months improved significantly after rhinoplasty. The kind of surgical approach and nasal procedures had no influence on post-operative satisfaction scores. However, patients with lower literacy were more satisfied with the procedure.

Rhinoplasty surgery significantly improved patient quality of life regarding nose function and appearance.

## Conflicts of interest

The authors declare no conflicts of interest.
